# TME3/347: A Teledentistry Consultation System and Continuing Dental Education via Internet

**DOI:** 10.2196/jmir.1.suppl1.e110

**Published:** 1999-09-19

**Authors:** L Alipour-Rocca, V Kudryk, T Morris

**Affiliations:** ^1^Telemedicine & Advanced Technology Research CenterFrederickUSA

**Keywords:** Dental Informatics, Teledentistry, Dentistry, Internet, Education

## Abstract

**Introduction:**

A Web-based teledentistry consultation system has been developed for the US Department of Defence dental clinics at various sites in Europe and the United States. This system is being used in over 55 dental clinics in Europe and 18 US Army dental clinics in the US. The system enables referring dentists to send consults, including dental images or radiographs to specialists, via Internet. Specialists review the consults and provide the diagnosis and treatment. This paper focuses on the implementation of this system, and focuses on the various areas in dentistry where this system is being currently utilized. The emphasis of this project has been on development of a scalable and expandable Internet-based teledentistry consultation system. The results of this study will expand the knowledge base of the technologies and clinical practices required changing the needs of modern, military dental care systems. One of the goals of the teledentistry project is to increase patient access to quality dental care. The other goal is to establish a cost effective teledentistry system.

**Methods:**

A teledentistry workstation consists of a laptop, a digital camera and requires Internet access. The referring dentist logs into a secure Web server, fills in the patient's demographics, chief complaints, provisional diagnosis information, attaches dental radiographs and sends it to the specialist of his choice. The specialist will receive an Email and the data is then sent to a database server. The specialist subsequently logs into the secure Web server and reviews the consult, radiographs and suggests his diagnosis and treatment.

**Results:**

The data collected on the database server shows an average of 40 consults per month. Currently, referrals to oral surgery have the highest number of consults, followed by prosthodontics and periodontics. An economic analysis has been conducted on the data that has been collected using this system. The results from this analysis show a return on investment within 21.5 months of deployment.

**Discussion:**

Videoconferencing, Web and Internet based technologies may be used to implement a user-friendly teledentistry solution. There are many advantages in using these technologies for retrieving dental information. Some of these advantages are:

- Ease of use and development
- Platform independence to the end user
- Cost-effectiveness

By providing dentists with easy, cost-effective access to specialists, it might be possible to improve the quality of care. This is achieved by facilitating the access to better and timely information to the dentists for better decision making and better communication between them and their patients. In the end, a telemedicine system is a tool for practising medicine: the science and art of healing.

